# Iso-α-acids, bitter components of beer, prevent obesity-induced cognitive decline

**DOI:** 10.1038/s41598-018-23213-9

**Published:** 2018-03-19

**Authors:** Tatsuhiro Ayabe, Rena Ohya, Keiji Kondo, Yasuhisa Ano

**Affiliations:** Research Laboratories for Health Science & Food Technologies, Kirin Company Ltd., Yokohama, Kanagawa 236-0004 Japan

## Abstract

Dementia and cognitive decline have become worldwide public health problems, and it was recently reported that life-style related diseases and obesity are key risk factors in dementia. Iso-α-acids, hop-derived bitter components of beer, have been reported to have various physiological functions via activation of peroxisome proliferator-activated receptor γ. In this report, we demonstrated that daily intake of iso-α-acids suppresses inflammations in the hippocampus and improves cognitive decline induced by high fat diet (HFD). Body weight, epididymal fat weight, and plasma triglyceride levels were increased in HFD-fed mice, and significantly decreased in iso-α-acids supplemented HFD-fed mice. HFD feeding enhances the production of inflammatory cytokines and chemokines, such as TNF-α, which was significantly suppressed by iso-α-acids administration. HFD-induced neuroinflammation caused lipid peroxidation, neuronal loss, and atrophy in hippocampus, and those were not observed in iso-α-acids-treated mice. Furthermore, iso-α-acids intake significantly improved cognitive decline induced by HFD-feeding. Iso-α-acids are food derived components that suppressing both lipid accumulation and brain inflammation, thus iso-α-acids might be beneficial for the risk of dementia increased by obesity and lifestyle-related diseases.

## Introduction

As the aged population increases, the incidence of dementia and cognitive decline has been growing rapidly, causing a worldwide public health problem. It was recently reported that the risk of dementia strongly correlates with levels of life-style related disease and obesity^1–5^. Epidemiological studies have indicated a negative correlation between body mass index (BMI) and cognitive function in healthy adults and Alzheimer’s disease patients^6^. A clinical trial suggested that BMI negatively correlates with the volumes of several brain regions, including the hippocampus^7^. Since definitive treatment for dementia has not been developed, preventing life-style related diseases and obesity would be an effective approach for prevention of dementia. However, there have been very few reports on the effects of food derived components on preventing dementia in the view point of suppressing obesity.

Hops (*Humulus lupulus* L.) have been used as the main ingredient of beer in order to add bitterness and flavor. Iso-α-acids are the main components of the bitterness in beer. Our group has previously demonstrated that iso-α-acids prevented dyslipidemia and type 2 diabetes in a diet-induced obese rodent model^8–10^, and improved glucose metabolism and decreased body fat in a clinical trial^11^. Furthermore, our group recently demonstrated that intake of iso-α-acids suppressed inflammation in the brain induced by amyloid β accumulation and prevented cognitive decline in Alzheimer’s disease model mice^12^. The Alzheimer’s disease model mice display cognitive decline accompanied by massive accumulation of amyloid β and inflammation. Iso-α-acids induce microglia into anti-inflammatory phenotype, suppressing inflammation and removing amyloid β via peroxisome proliferator-activated receptor γ (PPAR-γ) activation. However, it has not been elucidated whether iso-α-acids suppress the inflammation in brain induced by factors other than amyloid β, such as high fat diet-induced obesity. Since iso-α-acids have both anti-obesity and anti-dementia effects, it is expected that iso-α-acids would exhibit marked cognitive improving effects in obesity-induced cognitive impairment.

In the present study, we investigated whether iso-α-acids could suppress neuroinflammation in the brain, induced by a high fat diet-feeding. In addition, we examined whether iso-α-acids could improve cognitive function in obese mice, using behavioral pharmacological procedures.

## Results

### Dietary intake of iso-α-acids prevents high fat diet-induced obesity

**Figure 1:**
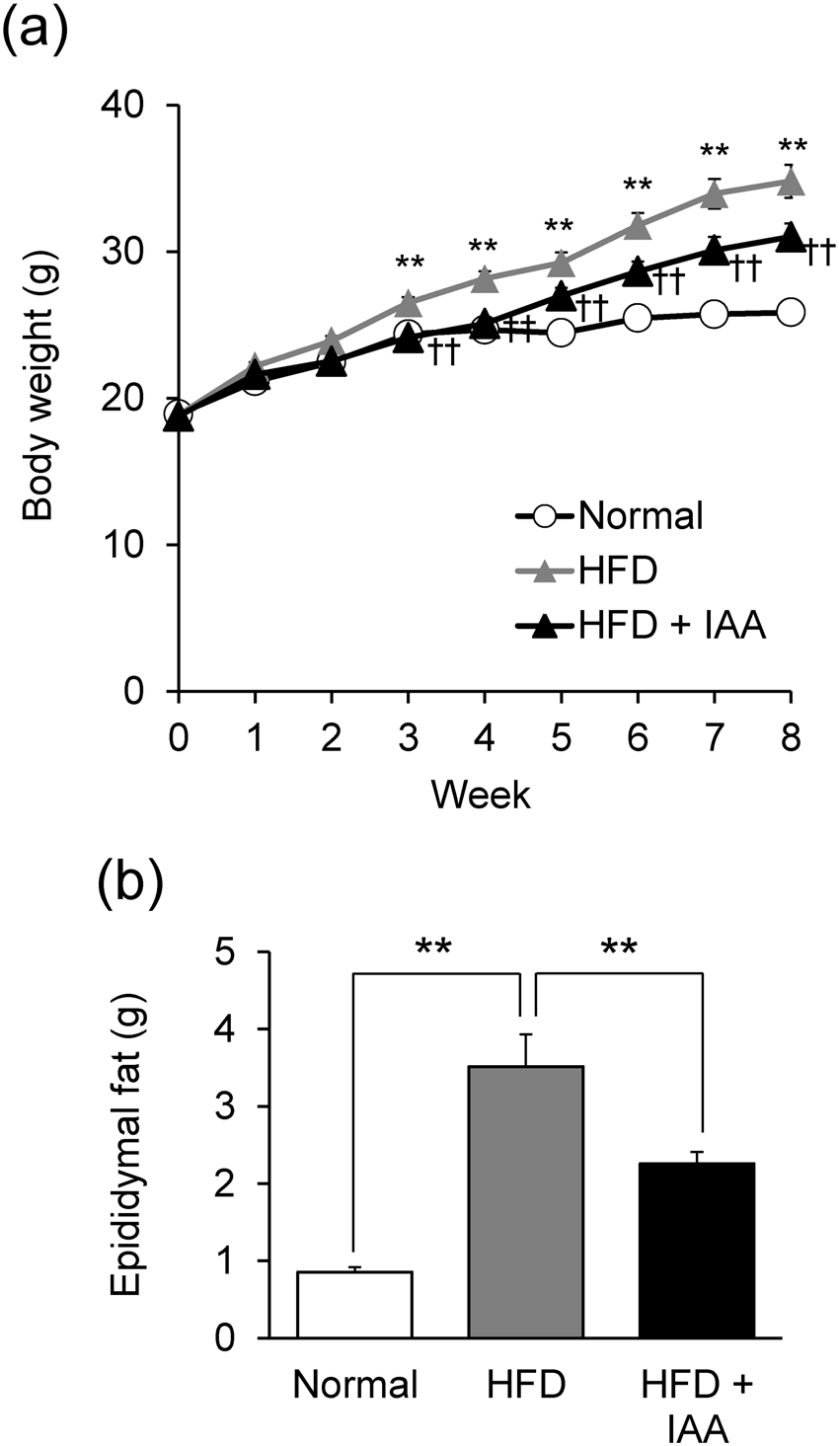
Dietary intake of iso-α-acids prevent excess body weight and adipose tissue gain induced by high fat diet intake. Male C57BL/6 J mice were fed either a normal diet, high fat diet (HFD), or HFD with 0.05% (w/w) iso-α-acids (IAA) supplementation for 8 weeks. (**a**) Body weight was measured every week. (**b**) Epididymal fat weight at the eighth week of feeding, when mice were sacrificed. All values are expressed as means ± SEM (n = 10 mice per group). (**a**) **P < 0.01 versus ND fed group, ^††^P < 0.01 versus HFD fed group. (**b**)**P < 0.01, *P < 0.05 versus each group.

To investigate the anti-obesity effects of iso-α-acids, mice were fed either a normal diet (ND), high fat diet (HFD), or HFD containing iso-α-acids (HFD + IAA) for 8 weeks. The body weight of HFD-fed mice gradually increased compared with ND-fed mice, and dietary intake of iso-α-acids lowered the body weight gain compared with HFD-fed mice, which showed significant difference between each group after 3 weeks of feeding (Fig. 1a). At the eighth week of feeding, epididymal fat weight of HFD-fed mice was significantly increased compared with ND-fed mice and the weight was lowered by iso-α-acids dietary administration (Fig. 1b). We then measured the plasma metabolic parameters: HFD feeding increased several parameters in plasma, such as aspartate aminotransferase (AST), urea nitrogen, and triglyceride, and iso-α-acids supplementation ameliorated those increases. Alanine aminotransferase (ALT), phospholipase, and the triglyceride levels in HFD + IAA-fed mice were significantly lowered compared with HFD mice (Table 1).

**Table 1:** Iso-α-acids attenuate high fat diet induced dyslipidemia.

	ND	HFD	HFD + IAA
TP	5.12 ± 0.08	5.40 ± 0.14	5.20 ± 0.08 g/dL
ALB	3.60 ± 0.06	3.92 ± 0.05	3.60 ± 0.06 g/dL
AST	59.6 ± 3.6	82.1 ± 3.0*	75.8 ± 7.3 IU/L
ALT	21.7 ± 2.4	26.2 ± 1.4	20.0 ± 2.0^†^ IU/L
ALP	202.4 ± 12.6	201.1 ± 9.9	203.9 ± 14.3 IU/L
UN	29.2 ± 1.0	34.4 ± 1.9*	30.4 ± 1.2 mg/dL
CRE	0.10 ± 0.02	0.12 ± 0.01	0.11 ± 0.02 mg/dL
PL	313.2 ± 20.9	365.2 ± 18.9	310.4 ± 14.7^†^ mg/dL
TG	61.2 ± 4.5	108.4 ± 11.2**	73.6 ± 11.0^†^ mg/dL
CHO	173.6 ± 14.2	190.8 ± 11.4	168.0 ± 12.3 mg/dL
GUL	292.8 ± 11.2	327.2 ± 10.7	308.8 ± 16.7 mg/dL

The plasma composition was analyzed using a biochemistry autoanalyzer. TP; total protein, ALB; albumin, AST; aspartate aminotransferase, ALT; alanine aminotransferase, alkaline phosphatase, UN; urea nitrogen, CRE; creatine, PL; phospholipase, TG; triglyceride, CHO; cholesterol, GUL; glucose. All values are expressed as means ± SEM (n = 10 mice per group). **P < 0.01, *P < 0.05 versus ND fed group, ^†^P < 0.05 versus HFD fed group.

### Iso-α-acids attenuate neuroinflammation and lipid peroxidation induced by HFD-feeding

**Figure 2:**
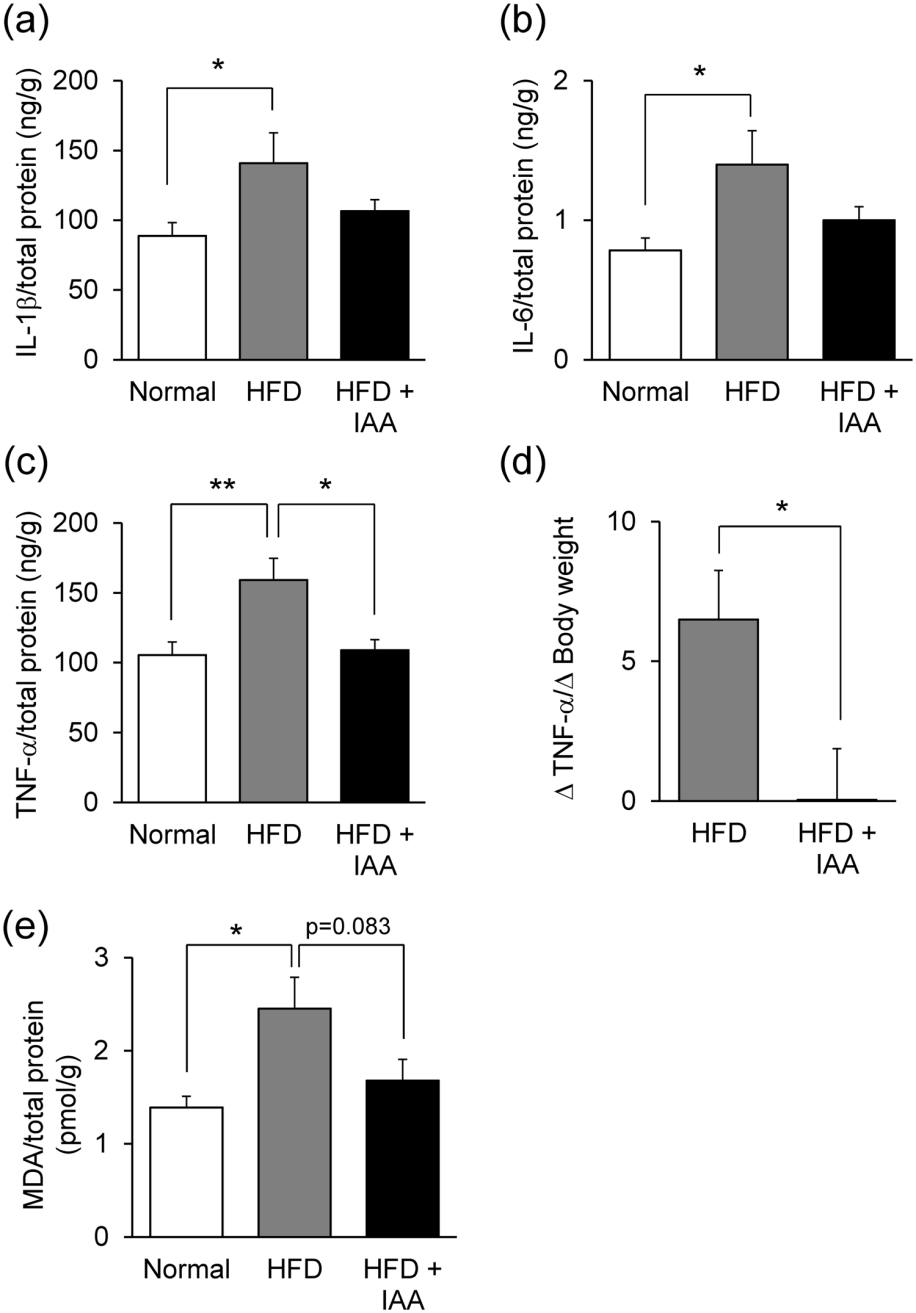
Iso-α-acids suppress obesity-induced neuroinflammation and lipid peroxidation in the hippocampus. Left hippocampus of ND-, HFD-, and HFD supplemented with iso-α-acids- fed mice were homogenated in TBS buffer and centrifuged supernatants were collected at the eighth week. (**a**–**c**) The levels of IL-1β (**a**), IL-6 (**b**) and TNF-α (**c**) were measured using a multi-plex assay. (**d**) The ratio of variation of the TNF-α level to that of body weight gain was calculated by the following formula: (individual TNF-α concentration minus average TNF-α concentration in ND-fed mice)/(individual body weight minus average body weight in ND-fed mice). (**e**) The level of malondialdehyde (MDA) in the hippocampus was measured by ELISA. All values are expressed as means ± SEM (n = 10 mice per group). **P < 0.01, *P < 0.05 versus each group.

Inflammatory cytokines and chemokines in the hippocampus are the key components of cognitive decline induced by obesity^13–16^. Thus, the effect of iso-α-acids on neuroinflammation was investigated by quantifying inflammatory cytokines and chemokines in the hippocampus using a Bio-Plex assay system. The levels of IL-1β, IL-6 and TNF-α were significantly elevated in HFD-fed mice compared with ND-fed mice. Dietary intake of iso-α-acids suppressed the elevation of IL-1β and IL-6 levels in the HFD-fed mice, and the TNF-α level was significantly reduced in the HFD + IAA-fed mice compared with HFD-fed mice (Fig. 2a,b,c, respectively). In order to evaluate the suppressive effects of iso-α-acids on neuroinflammation caused by HFD, the ratio of TNF-α increase to body weight gain versus control ND-fed group was compared between the HFD + IAA-fed mice and the HFD-fed mice. This index was significantly lower in HFD + IAA-fed mice compared with HFD-fed mice (Fig. 2d).

Previous reports indicated that TNF-α induces the production of reactive oxygen following lipid peroxidation, which causes cell membrane injury, leading to neurodegradation^17,18^. To evaluate the effects of iso-α-acids on lipid peroxidation, the level of malondialdehyde (MDA), the major product of lipid peroxidation, was measured. The level of MDA was significantly higher in HFD-fed mice compared with ND-fed mice, which was suppressed in the HFD + IAA-fed mice (Fig. 2e). The suppression of TNF-α by iso-α-acids supplementation might contribute to a reduction in lipid peroxidation.

### Iso-α-acids attenuate HFD induced hippocampal atrophy

**Figure 3:**
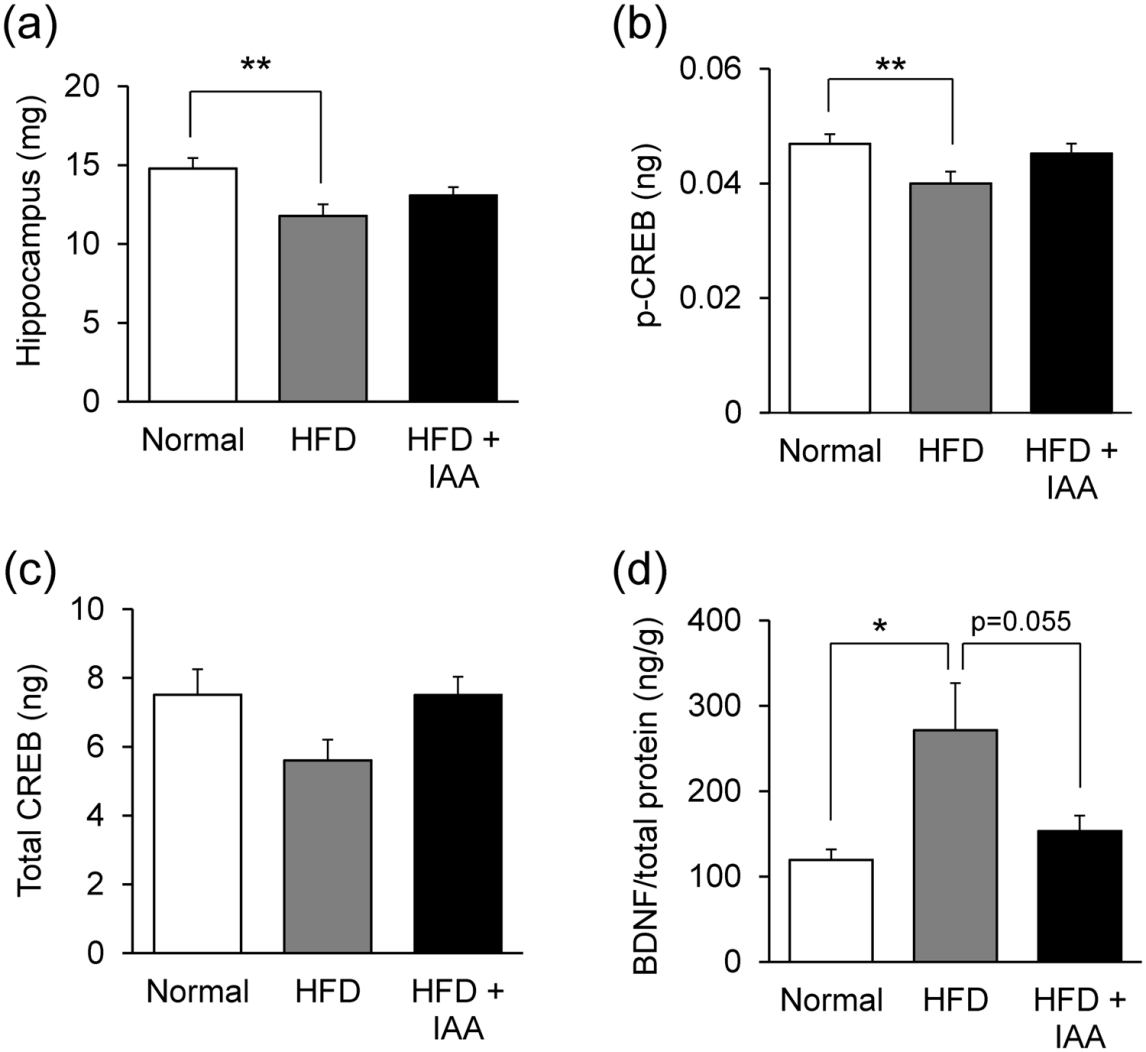
High fat diet loading induces atrophy and neurodegradation, which was suppressed by iso-α-acids supplementation. (**a**) Weights of hippocampus from ND-, HFD-, and HFD supplemented with iso-α-acids- fed mice, at the eighth week of feeding. (**b**–**d**) The levels of phosphorylated CREB (**b**), total CREB (**c**), and BDNF (**d**) in the hippocampus, which are regarded as indicators of neuronal cells. All values are expressed as means ± SEM (n = 10 mice per group). **P < 0.01, *P < 0.05 versus each group.

Previous reports suggested a correlation between obesity level and hippocampal volume^7^. Hippocampal weight was significantly lighter in HFD-fed mice compared with ND-fed mice and the weight was not changed in HFD + IAA-fed mice (Fig. 3a). To confirm hippocampal atrophy, the levels of cAMP response element binding protein (CREB) and phosphorylated CREB (p-CREB) were measured as parameters of neuronal activity^19,20^. The amount of p-CREB in HFD-fed mice was significantly lower compared with ND-fed mice; iso-α-acids administration prevented the down-regulation of p-CREB (Fig. 3b). The level of total CREB did not change significantly (Fig. 3c). The HFD-induced reduction of p-CREB may suggest that HFD feeding causes hippocampal atrophy. Furthermore, we investigated the level of brain derived neurotropic factor (BDNF), which plays an important role in neurogenesis^21,22^. Surprisingly, the level of BDNF was elevated in HFD-fed obese mice compared with ND- fed mice and the elevation was canceled by supplementation of iso-α-acids (Fig. 3d). It is assumed that the increase in BDNF is a response to improve hippocampal atrophy induced by HFD-feeding.

### Dietary intake of iso-α-acids improves obesity-induced cognitive decline

**Figure 4:**
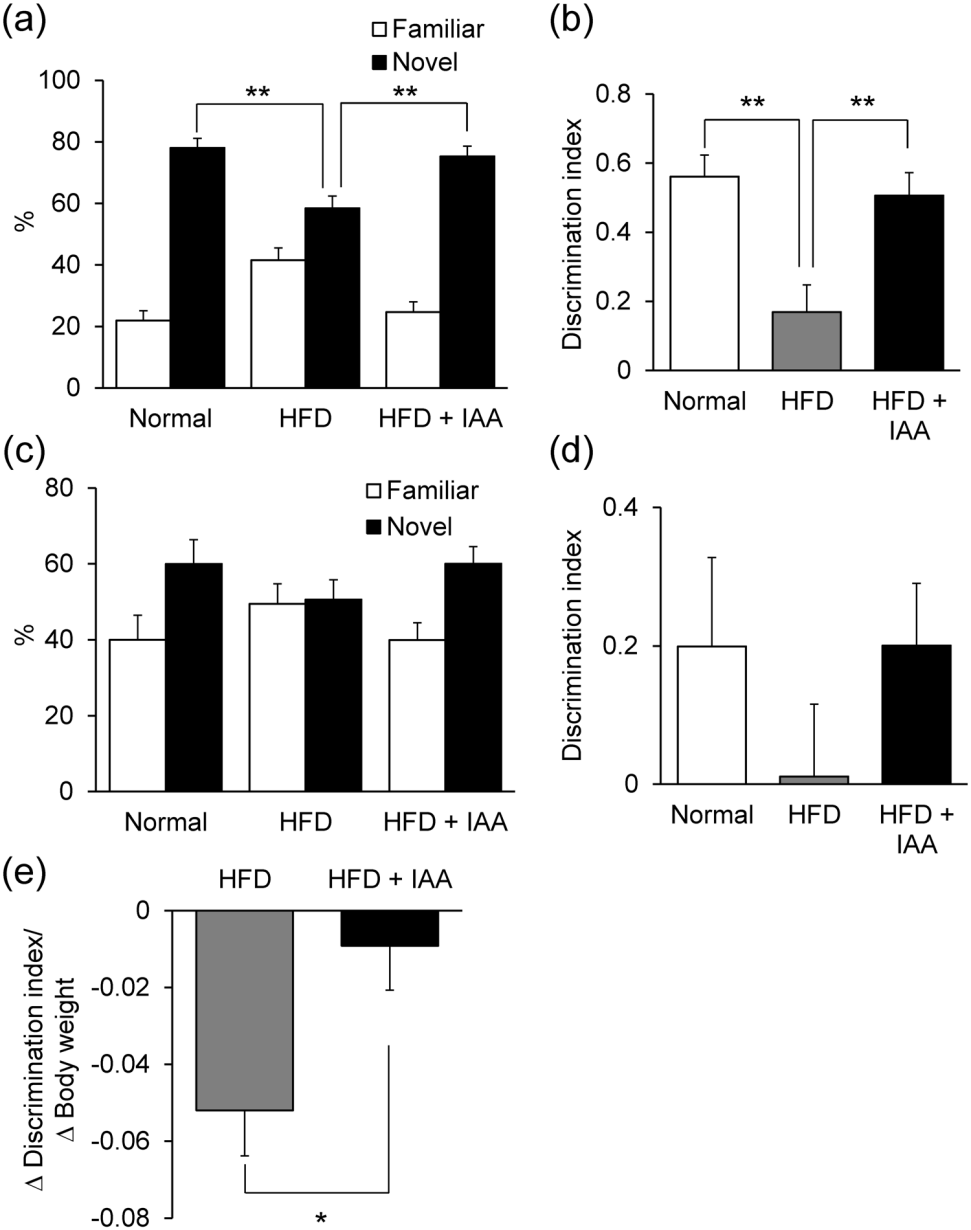
The anti-obesity effect of iso-α-acids leads to prevention of obesity-induced cognitive decline. The object recognition test (ORT; **a**,**b**) and object location test (OLT; **c**,**d**) were conducted at the seventh week. (**a**) Time spent in exploring novel or familiar objects over 5 min in the ORT. (**b**) Discrimination index (DI; (time spent investigating novel object minus time spent investigating familiar object)/(total exploration time)). (**c**) Time spent exploring novel or familiar locations over 8 min in the OLT. (**d**) Discrimination index. All values are expressed as means ± SEM (n = 8–10 mice per group). (**e**) The ratio of the DI score variation to the body weight gain was calculated by the following formula: (individual DI score minus average DI score in ND-fed mice)/(individual body weight minus average body weight in ND-fed mice). **P < 0.01, *P < 0.05 versus each group.

The cognitive functions, learning and memory function, were evaluated by behavioral pharmacology using an object recognition test (ORT) and object location test (OLT). Since a deficit in episodic memory is a typical pathology of amyloid β induced neuroinflammation, we first performed ORT. In the ORT, the time spent approaching the novel object was significantly reduced in HFD-fed mice compared with ND-fed mice, and was significantly extended in HFD + IAA-fed mice compared with HFD-fed mice (Fig. 4a). The discrimination index (DI) score was also significantly reduced in HFD-fed mice compared with the control mice and improved in HFD + IAA-fed mice (Fig. 4b). In OLT, the DI score in HFD-fed mice was lower compared with that of ND-fed mice and HFD + IAA-fed mice, though there were no significant differences between each group (Fig. 4d). The total approaching time and the distance mice moved in the ORT and OLT were not changed among these groups (data not shown), indicating that the dietary intake of iso-α-acids suppressed the cognitive decline induced by obesity. In order to evaluate the suppressive effects of iso-α-acids on cognitive dysfunction caused by HFD, the ratio of reduction of DI scores to body-weight gain versus control ND-fed group was compared between the HFD + IAA-fed mice and the HFD-fed mice. This index was significantly higher in HFD + IAA-fed mice compared with HFD-fed mice (Fig. 4e).

## Discussion

In the present study, we demonstrated that intake of iso-α-acids significantly reduced neuroinflammation induced by HFD feeding, in particular, the production of TNF-α was reduced in HFD + IAA-fed mice compared with HFD-fed mice. The cognitive impairment in obese mice was concomitantly relieved with the suppression of inflammation. Since it is difficult to cure dementia after its onset, preventative approaches have been explored. Chronic inflammation in the brain induces the pathology of dementia and various neurodegenerative disorders. Inflammation is induced by, not only amyloid β, but also physical conditions such as obesity, enterobacteria and stress, resulting in cognitive decline^23–26^. Recently, we demonstrated that dietary intake of iso-α-acids prevented inflammation in the brain and cognitive decline induced by amyloid β in Alzheimer’s disease model mice^12^. In our unpublished observation, iso-α-acids treatment rescued LPS induced cognitive impairment. The present study revealed that iso-α-acids also suppress inflammation induced by a high fat diet rich in saturated fatty acids. The increase of inflammatory cytokine TNF-α in brain by HFD-feeding was significantly suppressed by supplemental feeding of iso-α-acids (Fig. 2c). The ratio of TNF-α increase against body weight gain in the HFD + IAA-fed group was significantly lower compared with those in HFD-fed group (Fig. 2d), indicating that brain inflammation level in iso-α-acids treated mice is much lower than expected from its body weight gain by HFD feeding. This result suggests that iso-α-acids intake attenuates HFD-induced inflammation in the brain, not only by an anti-obesity effect but also by an anti-inflammatory effect. We suggest it would be more effective to trial the use of iso-α-acids to reduce the risk of dementia increased by obesity rather than any factor showing only anti-obesity activity.

We previously reported a potential mechanism for the anti-obesity and anti-inflammatory activities of iso-α-acids, which is that they bind and activate both peroxisome proliferator-activated receptor alpha (PPARα) and gamma (PPARγ)^8–10,12^. In this study, iso-α-acids supplementation reduced body weight gain and epididymal fat weight (Fig. 1), and improved several metabolic parameters such as AST, ALT, urea nitrogen, phospholipase, and triglycerides (Table 1), Since it is reported that activation of PPARs improve these circulating factors^27–29^, iso-α-acids might have protective effects on internal organs damaged in obesity. Previous reports suggested that activation of PPARα prevents amyloid β stimulated proinflammatory responses^30^, and activation of PPARγ suppresses inflammation in the brain in Alzheimer’s disease model mice^31–33^. Thus, iso-α-acids could be valuable for prevention of dementia in various pathways because of the dual agonistic effect on these receptors. Rosiglitazone is a potent agonist of PPARγ, and is used to treat type 2 diabetes^34–36^. A previous study reported that long term administration of rosiglitazone in HFD-induced obese rats improved spatial learning by correcting peripheral insulin resistance^37^. However, since rosiglitazone is used as a pharmaceutical agent, it is difficult to intake daily for prevention. To our knowledge, this is the first report that shows a food-derived PPARγ agonist, suitable for daily intake, improve HFD-induced inflammation in the brain and cognitive impairment.

Hippocampal lipid peroxidation, atrophy and a reduction in pCREB were observed in HFD-fed mice, however, this was not observed in mice fed HFD combined with iso-α-acids (Fig. 3). Previous reports suggested that oxidative stress and lipid peroxidation are associated with HFD-induced inflammation, which cause neuronal loss^13–18^. Since a deficit in episodic memory is a typical pathology of amyloid β induced neuroinflammation and neuronal loss, we studied the cognitive functions by behavioral pharmacology using ORT and OLT (Fig. 4). The tests revealed that the DI scores were reduced by HFD feeding and improved by adding iso-α-acids to HFD, indicating that intake of iso-α-acids significantly improved cognitive decline induced by HFD-feeding. The ratio of DI score decline against body weight gain in the HFD + IAA-fed group was significantly higher compared with that in HFD-fed group (Fig. 4e). This result indicates that the DI score in the HFD + IAA-fed mice was recovered to the same level of that in the ND-fed mice, although the body weight of HFD + IAA-fed mice is still higher than that of ND-fed mice, which may be induced by potent anti-inflammatory effect of iso-α-acids.

Iso-α-acids are bitter components of beer derived from hops and have been consumed for over a thousand years. Popular beer contains iso-α-acids at a concentration of 20–40 mg/L and some bitter beers, such as India Pale Ale, contains 50–80 mg/L. In our previously reported clinical trial, intake of iso-α-acids between 32 and 48 mg/day decreased body mass index, fasting blood glucose, and HbA1c^11^. Though we have not yet examined the cognitive improvement effect of iso-α-acids on humans, dietary intake of iso-α-acids might contribute to an improvement in cognitive function and prevent cognitive decline in obese people via suppression of inflammation in the brain and anti-obesity effects.

In conclusion, we demonstrated that iso-α-acids, bitter components of beer, prominently suppress inflammation in the hippocampus induced by intake of high fat diet and improve hippocampal atrophy and learning and memory functions. Notably, iso-α-acids display obesity suppressing activity as well as suppressing inflammation in the brain, which are little reports of food materials. Iso-α-acids are expected to decrease the risk of dementia and metabolic syndrome.

## Materials and Methods

### Materials

Isomerized hop extract (IHE) was purchased from Hopsteiner (Mainburg, Germany). IHE was an aqueous solution of iso-α-acids as a state of potassium salt, thus it was used as a source of iso-a-acids. The content of IHE was analyzed and described previously^12^. Briefly, IHE contains 30.5% (w/v) iso-α-acids, comprised of *trans*-isocohumulone (1.74% w/v), *cis*-isocohumulone (7.61% w/v), *trans*-isohumulone (3.05% w/v), *cis*-isohumulone (14.0% w/v), *trans*-isoadhumulone (0.737% w/v), and *cis*-isoadhumulone (3.37% w/v).

### Animals

Male 5 weeks old C57BL/6 J mice were purchased from Charles River Japan Inc. (Tokyo, Japan). Mice were maintained at room temperature (23 ± 1 °C) under constant 12-h light/dark cycles (light period from 8:00 am to 8:00 pm). All mice were acclimatized by feeding a standard rodent diet, CE-2 (Clea Japan, Tokyo, Japan), for 1 week. After acclimatization, mice were divided into three experimental groups: normal diet fed group (ND group), high fat diet (HFD) fed group (HFD group), and HFD plus 0.05% (w/w) iso-α-acids group (HFD + IAA group). This dosage of iso-α-acids was previously described to exhibit anti-inflammation effect in Alzheimer’s disease model 5xFAD mice^12^. The HFD + IAA and HFD group mice were fed 60 kcal% HFD (D12492, Research Diets Inc., New Brunswick, USA) with or without iso-α-acids supplementation, respectively. The treatment period was eight weeks, which was considered to be sufficient to cause HFD-induced neuroinflammation. We confirmed that iso-α-acids supplementation has no effect on food intake. For the ND group, a 10 kcal% fat containing diet (D12450J, Research Diets) was fed. The formulation and composition of HFD and ND were described in Table 2. Body weight was measured every week. All animal care and experimental procedures were done in accordance with the guideline of the Animal Experiment Committee of Kirin Company Ltd., and all efforts were made to minimize suffering. This study was approved by the Animal Experiment Committee of Kirin Company Ltd., and the approval ID was AN10266-Z00.

**Table 2:** High fat diet and normal diet formulation.

	HFD (D12492)		ND (D12450J)	
	g	kcal	g	kcal
Protein (%)	26	20	19	20
Carbohydrate (%)	26	20	67	70
Fat (%)	35	60	4	10
Total	100	100 kcal/g	5.24	3.85
Casein	200	800	200	800
L-cystine	3	12	3	12
Corn starch	0	0	506	2025
Maltodextrin 10	125	500	150	500
Sucrose	68.8	275	68.8	275
Cellulose, BW200	50	0	50	0
Soybean Oil	25	225	25	225
Lard	245	2205	20	180
Mineral Mix S10026	10	0	10	0
DiCalcium Phosphate	13	0	13	0
Calcium Carbonate	5.5	0	5.5	0
Potassium Citrate	16.5	0	16.5	0
Vitamin Mix V10001	10	40	10	40
Choline Bitartrate	2	0	2	0

### Analysis of metabolic parameters

To evaluate the anti-obesity effects of iso-α-acids on HFD-fed mice, mice were sacrificed at the eighth week of feeding (13 weeks old). Blood samples were collected from the heart into heparin-coated tubes and centrifuged at 3,000 × g for 5 min. Supernatants were collected as plasma samples, and the plasma composition was analyzed using a biochemistry autoanalyzer (7180 Automatic Analyzer, Hitachi, Tokyo, Japan). Epididymal fat and liver were also collected and weighed.

### Brain sample preparation and analysis

To evaluate the effect of iso-α-acids on the brain function of HFD loaded mice, the left hippocampus was collected and weighed. Samples were homogenized using a multi-beads shocker (Yasui Kikai, Osaka, Japan) in TBS buffer containing a protease inhibitor cocktail (BioVision, Mountain View, USA). The homogenates were centrifuged (50,000 × g, 30 min) and the supernatants were used in the following assays: total protein content of the supernatant was measured using the BCA protein assay kit (Thermo Scientific, Rockford, USA). Inflammatory cytokines and chemokines were evaluated using the Bio-Plex assay system (Bio-Rad, Richmond, USA). Lipid peroxidation was assessed by measuring malondialdehyde (MDA) using a MDA adduct competitive ELISA kit (Cell Biolabs, San Diego, USA). As an indicator of neuronal cells, the amounts of total CREB and phosphorylated CREB were measured using a CREB (total) ELISA kit (Invitrogen, Carlsbad, USA) and Phospho-CREB (S133) ELISA kit (R&D Systems, Minneapolis, USA), respectively. Brain derived neurotropic factor (BDNF) levels were evaluated using a BDNF ELISA kit (Promega, Madison, USA).

### Object recognition test (ORT) and object location test (OLT)

Cognitive function was evaluated in the seventh week of test diet feeding (12 weeks old), using two behavioral pharmacological procedures: object recognition test (ORT) and object location test (OLT). The ORT was performed as previously described^12^, with slight modifications. The experimental apparatus used in this study was a square open field (40 cm × 40 cm × 40 cm) made of grey polyvinyl chloride. The box was placed in a sound-isolated experimental room. Two pairs of wooden blocks were used as objects: two triangle prisms and two square pyramids. Each object was placed in a corner on the same side as the matching shape. The ORT was comprised of two periods: an acquisition period and a recall period. Before the acquisition period started, mice were moved into the experimental room for at least 16 h. Each mouse was then placed into the experimental apparatus in the presence of two objects, and allowed to explore freely for 10 min. After 24 h, the recall period was performed. At the recall period, one object of each pair was replaced with a novel object (wooden white sphere). Each mouse was again placed into the apparatus for 5 min, and the exploration time with the familiar object and novel object was measured. The discrimination index was calculated using the following formula: (novel object exploration time minus familiar object exploration time)/(total exploration time).

In the OLT, visual cues (black, white, or stripe-pattern picture) were placed on the wall of the square open field, so that mice could distinguish each direction. Brown glass vials were used as objects. The OLT was comprised of three periods: a habituation period, an acquisition period, and a recall period. The habituation period was started 24 h after the ORT was completed. Each mouse was placed into the experimental apparatus without any objects for 10 min. The acquisition period was performed 24 h after the habituation period. In the acquisition period, each mouse was again placed into the apparatus with two objects at the corners on the same sides for 5 min. After 4 h, the recall period was performed. Each mouse was reintroduced into the apparatus with two objects at the two diagonal corners for 8 min. Similar to the ORT, the exploration time with the familiar object and novel object was measured, and the discrimination index was calculated.

### Statistical analysis

All values are expressed as means ± SEM. Body weight changes were analyzed by two-way ANOVA comparing diets and time effects. Two-group comparisons were analyzed by student’s *t* test. All other experimental data were analyzed by one-way ANOVA, followed by Tukey-Kramer’s test. P < 0.05 was considered statistically significant.

## References

[1] Vanhanen M (2006). Association of metabolic syndrome with Alzheimer disease. Neurology..

[2] Whitmer RA (2008). Central obesity and increased risk of dementia more than three decades later. Neurology..

[3] Xu WL (2011). Midlife overweight and obesity increase late-life dementia risk. Neurology..

[4] Ohara T (2011). Glucose tolerance status and risk of dementia in the community. Neurology..

[5] Albanese E (2017). Body mass index in midlife and dementia: Systematic review and meta-regression analysis of 589,649 men and women followed in longitudinal studies. Alzheimers. Dement..

[6] Waldstein SR, Katzel LI (2006). Interactive relations of central versus total obesity and blood pressure to cognitive function. Int. J. Obes..

[7] Raji CA (2010). Brain structure and obesity. Hum. Bra. Map..

[8] Yajima H (2004). Isohumulones, bitter acids derived from hops, activate both peroxisome proliferator-activated receptor α and γ and reduce insulin resistance. J. Biol. Chem..

[9] Yajima H (2005). Prevention of diet-induced obesity by dietary isomerized hop extract containing isohumulones, in rodents. Int. J. Obes..

[10] Miura Y (2005). Dietary isohumulones, the bitter components of beer, raise plasma HDL-cholesterol levels and reduce liver cholesterol and triacylglycerol contents similar to PPARα activations in C57BL/6 mice. Br. J. Nutr..

[11] Obara K, Mizutani M, Hitomi Y, Yajima H, Kondo K (2009). Isohumulones, the bitter component of beer, improve hyperglycemia and decrease body fat in Japanese subjects with prediabetes. Clin. Nutr..

[12] Ano Y (2017). Iso-α-acids, bitter components of beer, prevent inflammation and cognitive decline induced in a mouse model of Alzheimer’s disease. J. Biol. Chem..

[13] Jeon BT (2012). Resveratrol attenuates obesity-associated peripheral and central inflammation and improves memory deficit in mice fed a high-fat diet. Diabetes..

[14] Zhang X, Dong F, Ren J, Driscoll MJ, Culver B (2005). High dietary fat induces NADPH oxidase-associated oxidative stress and inflammation in rat cerebral cortex. Exp. Neurol..

[15] Kapogiannis D, Mattson MP (2011). Disrupted energy metabolism and neuronal circuit dysfunction in cognitive impairment and Alzheimer’s disease. Lancet Neurol..

[16] Nerurkar PV (2011). Momordica charantia (bitter melon) attenuates high-fat diet-associated oxidative stress and neuroinflammation. J. Neuroinflammation..

[17] Butterfield DA, Lauderback CM (2002). Lipid peroxidation and protein oxidation in Alzheimer’s disease brain: Potential causes and consequences involving amyloid β-peptide-associated free radical oxidative stres. s. Free Radic. Biol. Med..

[18] Markesbery WR, Kryscio RJ, Lovell MA, Morrow JD (2005). Lipid peroxidation is an early event in the brain in amnestic mild cognitive impairment. Ann. Neurol..

[19] Lonze BE, Ginty DG (2002). Function of regulation of CREB family transcription factors in the nervous system. Neuron..

[20] Lee J (2005). Mitochondrial cyclic AMP response element-binding protein (CREB) mediates mitochondrial gene expression and neuronal survival. J. Biol. Chem..

[21] Pencea V, Bingaman KD, Wiegand SJ, Luskin MB (2001). Infusion of brain-derived neurotrophic factor into the lateral ventricle of the adult rat leads to new neurons in the parenchyma of the striatum, septum, thalamus, and hypothalamus. J. Neurosci..

[22] Scharfman H (2005). Increased neurogenesis and the ectopic granule cells after intrahippocampal BDNF infusion in adult rats. Exp. Neurol..

[23] Heneka MT (2015). Neuroinflammation in Alzheimer’s diseas. e. Lancet. Neurol..

[24] Patel NS (2005). Inflammatory cytokine levels correlate with amyloid load in transgenic mouse models of Alzheimer’s disease. J. Neuroinflammation..

[25] Almond MH, Edwards MR, Barclay WS, Johnston SL (2013). Obesity and susceptibility to severe outcomes following respiratory viral infection. Thorax..

[26] Chidiac C (2012). Pneumococcal infections and adult with risk factors. Med. Mal. Infect..

[27] Fernández-Miranda C (2008). A pilot trial of fenofibrate for the treatment of non-alcoholic fatty liver disease. Dig. Liver. Dis..

[28] Filippatos TD (2007). The effect of orlistat and fenofibrate, alone or in combination, on small dense LDL and lipoprotein-associated phospholipase A2 in obese patients with metabolic syndrome. Atherosclerosis..

[29] Balakumar P, Chakkarwar VA, Singh M (2009). Ameliorative effect of combination of benfotiamine and fenofibrate in diabetes-induced vascular endothelial dysfunction and nephropathy in the rat. Mol. Cell. Biochem..

[30] Combs CK, Bates P, Karlo JC, Landreth GE (2001). Regulation of β-amyloid stimulated proinflammatory responses by peroxisome proliferator-activated receptor α. Neurochem Int..

[31] Combs CK, Johnson DE, Karlo JC, Cannady SB, Landreth GE (2000). Inflammatory mechanisms in Alzheimer’s disease: Inhibition of β-amyloid-stimulated proinflammatory responses and neurotoxicity by PPARγ agonist. s. J. Neurosci..

[32] Heneka MT (2005). Acute treatment with the PPARγ agonist pioglitazone and ibuprofen reduces glial inflammation and Aβ1–42 levels in APPV717I transgenic mice. Brain.

[33] Fuenzalida K (2007). Peroxisome proliferator-activated receptor γ up-regulates the Bcl-2 anti-apoptotic protein in neurons and induces mitochondrial stabilization and protection against oxidative stress and apoptosis. J. Biol. Chem..

[34] The DREAM (Diabetes REduction Assessment with ramipril and rosiglitazone Medication) Trial Investigators. Effect of rosiglitazone on the frequency of diabetes in patients with impaired glucose tolerance or impaired fasting glucose: a randomised controlled trial. *Lancet*. **368**, 1096–1105 (2006). 10.1016/S0140-6736(06)69420-8 16997664

[35] Mayerson AB (2002). The effects of rosiglitazone on insulin sensitivity, lipolysis, and hepatic and skeletal muscle triglyceride content in patients with type 2 diabetes. Diabetes..

[36] Yang WS (2002). Synthetic peroxisome proliferator-activated receptor-γ agonist, rosiglitazone, increases plasma levels of adiponectin in type 2 diabetic patients. Diabetes Care..

[37] Pathan AR, Gaikwad AB, Viswanad B, Ramarao P (2008). Rosiglitazone attenuates the cognitive deficits induced by high fat diet feeding in rats. Eur. J. Pharmacol..

